# Optimizing Durability in Radiofrequency Ablation of Atrial Fibrillation

**DOI:** 10.19102/icrm.2021.120505

**Published:** 2021-05-15

**Authors:** Zain I. Sharif, E. Kevin Heist

**Affiliations:** ^1^Clinical Cardiac Electrophysiology Department, Massachusetts General Hospital, Boston, MA, USA

**Keywords:** Atrial fibrillation, lesion durability, radiofrequency ablation

## Abstract

Radiofrequency ablation (RFA) remains a highly effective therapy in the management of paroxysmal atrial fibrillation (PAF) and is an important therapeutic option in the management of persistent atrial fibrillation (PeAF) when clinically indicated. Lesion size is influenced by many parameters, which include those related to energy application (RFA power, temperature, and time), delivery mechanism (electrode size, orientation, and contact force), and the environment (blood flow and local tissue contact, stability, and local impedance). Successful durable RFA is dependent on achieving lesions that are reliably transmural and contiguous, whilst also avoiding injury to the surrounding structures. This review focuses on the variables that can be adjusted in connection with RFA to achieve long-lasting lesions that enable patients to derive the maximum sustained benefit from pulmonary vein isolation and additional lesion sets if utilized.

## Introduction

Radiofrequency (RF) ablation (RFA) remains a highly effective therapy in the management of paroxysmal atrial fibrillation (AF) (PAF) and is an important therapeutic option in the management of persistent AF (PeAF) when clinically indicated.

This review focuses on variables that can be adjusted in connection with RFA to achieve long-lasting lesions that enable patients to derive the maximum sustained benefit from pulmonary vein (PV) isolation (PVI) and additional lesion sets if created. For the purposes of this review, we will not discuss alternative ablation technologies or the use of strategies other than RFA PVI, focusing instead on the durability of lesion application.

## Physics of radiofrequency lesion application

RFA lesions are created via alternating current with a high-frequency range of 100 to 2,000 kHz, which generates a zone of resistive heating adjacent to the electrode tip and a more distal zone of conductive heating, with heat lost to circulating blood. The heating of the cardiac tissue is mainly resistive.

Lesion size is influenced by many parameters, including those related to energy application (RFA power, temperature, and time), delivery mechanism [electrode size, orientation, and contact force (CF)], and the environment (blood flow and local tissue contact, stability, and local impedance). Success with achieving durable RFA is dependent upon creating lesions that are reliably transmural and contiguous^1,2^ while simultaneously avoiding injury to the surrounding structures.

Myocardial tissue typically will be permanently destroyed at temperatures of approximately 55°C sustained for several seconds,^3^ with increased tissue heating leading to larger ablation lesions.^4^ Longer ablation durations allow for the zone of resistive heating to expand and facilitate conductive heating.

Power describes the rate at which energy is consumed per unit of time. The relationship between power and energy is represented by the following equation: energy (J) = power (W) × time (s). Therefore, the higher the power, the less the amount of time required to deliver equivalent energy for lesion formation. The implications of increasing the power with a shorter duration to deliver equivalent energy are described later.

## Lesion durability

### Optimal lesion characteristics for durability

PV reconnection (PVR) is a common cause of AF recurrence after PVI, often necessitating repeat ablation. PVR remains the most common finding in patients who experience AF recurrence.^5^ Increasing single procedure success is key early on in the disease process when considering the electrical and mechanical remodeling processes the atria undergo with prolonged time spent in AF,^6^ which makes rhythm control more challenging.^7^

Cardiac magnetic resonance imaging (MRI) studies demonstrate that acute PVI can be obtained by a combination of ablation-induced reversible (edema) and irreversible (necrosis) atrial injury.^8^ Achieving a lesion set encompassing necrosis rather than edema due to the transient nature of the latter appears key to the formation of a durable lesion set with no PVR. The presence of edema rather than irreversible necrosis can be suggested during ablation by the emergence of dormant conduction following the administration of adenosine or after a waiting period. By optimizing the lesion quality by virtue of stability, first-pass contiguity, and improving lesion geometry, as will be described later, irreversible durable necrotic lesions rather than reversible edema are promoted.

### Limitations in the assessment of durability

Limitations of studies examining the durability of lesion sets include that a repeat invasive procedure is necessary to evaluate for PVR as some patients maintain sinus rhythm after PVI despite PVR in some or even all PVs. This is challenging to pursue in large-scale studies, particularly when patients have had an acceptable clinical response to PVI. Patients lost to follow-up and underreporting of symptoms can also dilute the follow-up data.

As a general rule, the more rigorous the method of monitoring, the more AF that is detected.^9^ In the Cryoballoon vs. Irrigated Radiofrequency Catheter Ablation: Double Short- vs. Standard-exposure Duration (CIRCA-DOSE) study, one of the landmark PVI trials performed in recent years, implantable cardiac monitoring (ICM) devices were used for examining AF burden and revealed a greater than 98% reduction in AF burden in both the RFA and cryoablation arms. Examining AF burden via ICM when assessing the durability of outcomes is a more rigorous objective measure of long-term outcomes than the use of periodic electrocardiogram (ECG)/Holter monitoring, with rates of compliance with noninvasive rhythm monitoring falling as low as 59% in contemporary RFA studies.^10^ Symptoms do not tend to correlate with AF burden, with follow-up data from this study demonstrating a 21.6% to 26% difference between symptomatic and any AF/atrial flutter (AFL)/atrial tachycardia (AT).^11^ The inclusion of AFL and AT among the considerations during follow-up can cloud the durability of PVI lesion set assessment somewhat (although AT may be of a PV-gap reentrant nature), which is something to bear in mind while assessing the durability of the PVI lesion set based on subsequent arrhythmia detection. Non-PV triggers causing AF recurrence must also be considered when AF detection during rhythm analysis is used as a follow-up endpoint.

It is important to note (as highlighted by the most recent Heart Rhythm Society guidelines) that, when referring to lesion durability, that 50% of patients within the first three months of AF RFA experience an early recurrence of AF (defined as any recurrence of AF > 30 seconds during the first three months of follow-up^12^); however, this time frame can be referred to as the therapy stabilization phase or “blanking period,” with 50% of these recurrences also manifesting as late recurrences.^13^

### Energy delivery

**Figure 1:**
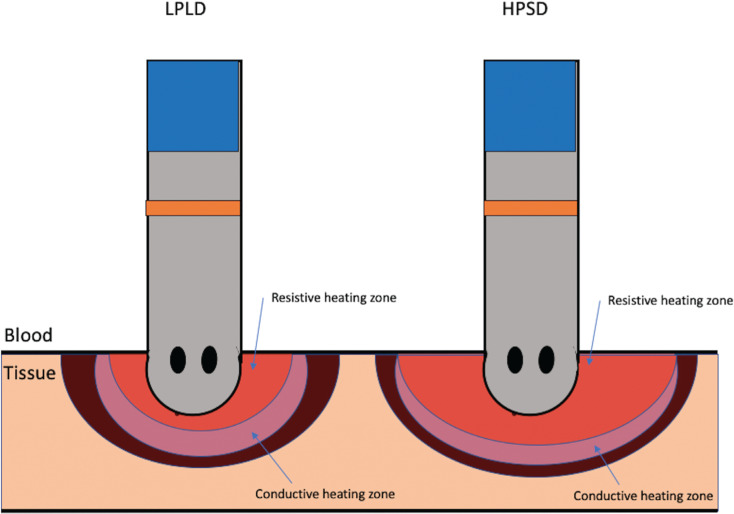
Comparison of different power and time strategies for lesion formation. HPSD: high-power, short-duration ablation; LPLD: low-power, long-duration ablation.

High-power, short-duration (HPSD) ablation appears to lead to more durable lesions with reduced tissue edema formation.^14^ From a biophysical standpoint, this appears to be related to a modification in the proportion of resistive and conductive heating zones; specifically, the radius of the resistive heating zones is increased, delivering immediate lethal energy to a larger tissue volume, with the surrounding conductive heating zone tapering off more quickly. In an ex vivo and in silico study comparing different ablation parameters, with HPSD ablation of 70 W at seven seconds at 15 to 20 g of CF relative to that of 30 W of power at 30 seconds and the same CF, the lesions were wider, particularly at the surface, although the lesion volume was similar.^15^ As the power increases above 50 to 60 W and ablation times become shorter, lesions become increasingly larger in diameter, although their depth decreases, which potentially has implications for thicker atrial tissue such as the mitral isthmus, the vein of the Marshall network, and the left atrial appendage orifice. Using this approach, another animal study demonstrated wider lesions, less reversible injury, more uniform lesions, and a continuous ablation lesion set with similar RF applications **(Figure 1)**.^16^

Among 51 patients with PAF or PeAF undergoing HPSD ablation with an irrigated CF-sensing catheter at 50 W and a CF of 10 to 40 g, those with PAF demonstrated a freedom from AF rate of 86% at both one and two years, while those with PeAF showed rates of 83% and 72% at one and two years.^17^ Surrogates for lesion creation in this study were loss of pacing capture and impedance drop. The follow-up consisted of three- and 12-month seven- and 14-day continuous monitoring. Meanwhile, three-year observational outcomes data from Bunch et al.,^18^ examining HPSD versus low-power, long-duration ablation in a total of 1,333 patients with AF (approximately half of whom were PeAF patients) revealed similar long-term outcomes at three years (recurrence rates of 12.9% vs. 16.2% and 26.5% vs. 30.7%, respectively, at one and three years). HPSD ablation consisted of 50 W of power for five to 15 seconds on the anterior wall and two to three seconds on the posterior wall. CF-sensing catheters were used where available. Of note, sheaths were not maintained in the left atrium (LA). Follow-up in this group consisted of ambulatory monitoring at three-month intervals postablation in the first year and then in cases with symptomatic recurrence thereafter.

Recent work by Yavin et al.^19^ compared HPSD and moderate-power, moderate-duration (MPMD) ablation with respect to lesion durability by measuring long-term clinical efficacy and procedural safety in a prospective, single-center study of 112 patients with PAF and PeAF undergoing first-time PVI plus or minus an additional lesion set relative to a historic cohort with MPMD ablation. Of note, all procedures were performed with jet ventilation and general anesthesia (GA). The Ablation Index (AI) (Biosense Webster, Diamond Bar, CA, USA) was not used due to its unavailability at the beginning of the study. HPSD ablation consisted of 45 to 50 W of power for eight seconds on the posterior wall and 15 seconds on the left PV ridge and septal aspect of the right PV. First-pass isolation was achieved in 90.2% in the HPSD group versus 83% in the MPMD group (p = 0.006), with similar rates of steam pop (0.07% vs. 0.03%; p = 0.18). Durability as assessed by the incidence of PVR at repeat study was significantly lower in the HPSD group (16.6% in 18 patients) than in the MPMD group (52.2% in 23 patients). Of the five PVRs in the HPSD group, all occurred in the septal aspect of the right PV or the anterior left PV. These rates should be viewed with caution, however, as PVR was only assessed where there was a clinical recurrence of AF; therefore, the absolute rate of PVR in all study participants remains unknown. The authors of this paper also expressed caution regarding catheter stability with this approach, with patients observed to have reconnection during redo procedures found retrospectively to have unstable catheter ablation during their index ablation (≥ 1 mm for ≥ 50% application duration). This is intuitive considering that a few seconds of instability may constitute, for example, half of the actual lesion duration in HPSD ablation. Therefore, CF and stability improvement such as with GA, steerable sheaths, and jet ventilation also play a role in this approach.

### Contact force

Achieving the capacity to measure CF between the catheter tip and the target tissue is a major milestone in optimizing ablation outcomes. Current catheters incorporating CF include the TactiCath™ (Abbott, Chicago, IL, USA) and Thermocool^®^ SmartTouch™ (Biosense Webster) catheters. With power-controlled ablation, there is a significant relationship between lesion size and tissue contact, with increased CF associated with larger and more transmural lesions^20^ but a greater risk of steam pop.^21^

CF-guided ablation appears to predict the durability of ablation. Acute success in wide antral circumferential ablation (WACA) with non-CF catheters is limited, with Andrade et al. in 2014 demonstrating that only 66% of PV circles are acutely adenosine-proof at the time of PVI as compared with 92% following CF-guided PVI in patients with PAF.^22^ In their study, the one-year off-antiarrhythmic freedom from recurrent atrial arrhythmia rates were 88% in the CF group and 66% in the non–CF-guided group (p = 0.047).

The TactiCath™ CF Ablation Catheter Study for AF (TOCCASTAR)^23^ and Thermocool^®^ SmartTouch™ Catheter for the Treatment of Symptomatic PAF (SMART-AF)^24^ study further demonstrated the superior durability of lesion-set application with CF-guided ablation. In TOCCASTAR, approximately 75.9% of PAF patients treated optimally with CF-guided ablation using the TactiCath™ catheter were free from AF at 12 months of follow-up, relative to only 58.1% of patients who did not receive 10 g or more of force. A trend existed toward a lower rate of repeat ablations in the optimal CF group (≥ 90% of the lesions created with a CF ≥ 10 g) as compared with in the nonoptimal CF group (7.2% vs. 16.1%; p = 0.078) and the control group (7.2% vs. 12.7%; p = 0.148). Multivariate analyses from the SMART-AF study with the Thermocool^®^ SmartTouch™ catheter demonstrated that investigators staying within their selected ranges of 80% or more during RFA were approximately 4.25 times more likely to achieve clinical success at 12 months when compared with those who did not [95% confidence interval (CI): 1.53–11.79].

Of studies examining PV durability with invasive PV assessment, the Randomized Trial of Two Different Strategies to Treat PAF (GAP-AF) and TactiCath^®^ Prospective Effectiveness Pilot Study (EFFICAS) studies, in particular, offer insight. GAP-AF, one of the earliest and largest studies that remapped PVs post-PVI in PAF, used irrigated catheters without CF and with patients under conscious sedation, achieving a durable PVI rate of only 30% at three months’ repeat study.^25^ Data of PeAF treatment using non–CF-guided catheters from the PVI-alone arm of the Outcome of AF Ablation After Permanent PV Antrum Isolation With or without Proven Left Atrial Posterior Wall Isolation (LIBERATE) study suggested a three-month durability rate of 60% (12/20 patients).^26^ Data from the EFFICAS 1 and 2 studies demonstrated increased durability was attained with CF in patients with PAF. A force–time integral (FTI) value of less than 400 gs during WACA was associated with PVR at three months in the initial EFFICAS 1 study. Meanwhile, the prospective multicenter EFFICAS 2 study showed 90% less veins with gaps (1.8% vs. 19%) after PVI with contiguous ablation lines and low catheter movement where CF guidelines were adhered to as compared with in patients in the EFFICAS 1 study, where these guidelines were not adhered to (p = 0.005).^27,28^ These guidelines were a target CF of 20 g (range: 10–30 g) and an FTI of at least 400 gram-seconds (gs). Patients underwent a PV remapping at three months to assess lesion durability.

A meta-analysis in 2015 of nine nonrandomized studies involving 1,148 patients examined CF-guided PVI versus non–CF-guided PVI, demonstrating a 37% relative risk reduction (p = 0.01) in AF recurrence post-PVI at a median of 12 months.^29^

The recent Prospective Review of the Safety and Effectiveness of the Thermocool^®^ SmartTouch™ Surround Flow Catheter Evaluated for Treating Symptomatic Persistent AF (PRECEPT) trial by Mansour et al.^30^ used CF-sensing irrigated catheters; over half of study participants received a lesion set beyond PVI. In 348 patients with PeAF, the rate of freedom from documented recurrence of AF/AFL/AT of 30 seconds or longer was 61.7% and that of freedom from symptomatic recurrences was 80.4% at 15 months. Additionally, 7.8% of patients underwent a redo ablation at 15 months. Monitoring included Holter monitoring at baseline and six-, 12-, and 15-month visits with transtelephonic monitoring occurring monthly or if symptoms recurred.

### Ablation index, force–time integral, and interlesion distance

Another advancement that has emerged in recent years in improving lesion durability has been the adoption of objective lesion representational tools such as the CARTO VISITAG™ Module with Ablation Index (Biosense Webster) and Lesion Index (Abbott), which provide visual lesion representation based on the integration of power, CF, stability, and time parameters. Both FTI and FTI/wall thickness as measured by computerized tomography have been previously demonstrated to be predictors of gap and acute dormant conduction.^31^

The CLOSE protocol was developed on the basis of a study by El Hadad et al., encompassing a point-by-point RFA approach aiming to isolate PVs with a contiguous, reproducible, and optimized lesion set. In the guiding study, the AI parameters of at least 400 or greater (posterior) or 550 or greater (anterior) with an interlesion distance (ILD) of 6 mm or less were associated with a 93% specificity to predict durable segments.^32^ This protocol was evaluated in a pilot study of 130 PAF patients with an average age of 59 years; here, acute durable PVI rates were high, with a 98% adenosine-proof isolation rate leading to impressive one-year outcomes. Freedom from documented AF/AT/AFL off antiarrhythmic drug (AAD) therapy was 91.3% and on AAD therapy was 96.2%. Follow-up in this study consisted of Holter monitoring at three, six (both 24 hours), and 12 months (seven days). Ten of 130 patients with documented recurrence in this study underwent repeat ablation. In six of the 10 patients, permanent isolation of veins was noted, while, in the remaining four patients, five gaps were noted in four of eight circles; four of these five gaps were explained by an ILD of greater than 6 mm, with the remaining gap demonstrating optimal parameters.

The Prospective Randomized Amlodipine Survival Evaluation (PRAISE) study incorporated an eight- to 10-week mandated repeat electrophysiology study in 40 patients undergoing CLOSE-protocol, AI-guided PVI for PeAF of less than 12 months in duration and no structural heart disease. PVI durability was noted in 78% of patients, with 93% of PVs remaining isolated. Interestingly, late reconnection was most commonly encountered in carinal regions, with a carinal line originally required for acute isolation in 25% of study participants.^33^

The results of the CLOSE to CURE study, where the AF burden was assessed by means of implantable cardiac monitors following CLOSE-protocol PVI in PAF patients, provided further insight into the durability of this approach. Atrial tachyarrhythmia burden decreased from 2.68% at baseline to 0% at both one and two years, with rates of single-procedure freedom from atrial tachyarrhythmia of 87% at one year and 78% at two years, respectively.^34^

Recent results of the Evaluation of AI and VISITAG™ (ABI-173) (VISTAX)^35^ trial, the first multicenter prospective study (n = 340) evaluating the effectiveness and safety of CLOSE protocol–guided RFA in patients with PAF, demonstrated a high rate of first-pass isolation (82.4%) with a rate of 12-month freedom from arrhythmia close to 80% and that of 12-month freedom from repeat ablation of 90.4%, with 14 of 34 patients undergoing repeat ablation demonstrating a durable PVI lesion set. The follow-up protocol consisted of 24-hour Holter monitoring at three, six, and 12 months and weekly and symptom-driven transtelephonic monitoring.

The contribution of ILD to durability was noted in the aforementioned study by El Hadad et al., where an ILD of 5 mm or less at index CF-guided PVI was found to be significantly associated with a low incidence of PVR.^25^ Recent randomized data suggest that targeting a maximal interlesion index of 3 to 4 mm rather than the original CLOSE protocol cutoff of 6 mm can increase acute procedural success, which may translate to increased durability. This study of 42 patients randomized to both approaches was terminated early due to the superiority of the shorter ILD protocol for first-pass PVI (90.9% vs. 35%; p < 0.0001) with a shorter procedure time.^36^

The combination of objection lesion representation parameters as described can be combined with other ablation strategies such as HPSD. As the formula for the AI incorporates CF, power, and time in a weighted formula, by increasing the power, a shorter time of ablation would be required for an equivalent lesion, with standard AI targets shown to have favorable results with this strategy.^37^

### First-pass isolation

First-pass PVI may predict long-term clinical success after CF-guided PVI for PAF. A recent real-world cohort study of 157 patients undergoing PVI with a CF of 10 to 20 g and an HPSD of 40 W reported improved one-year clinical outcomes (recurrent atrial tachyarrhythmia or freedom from repeat ablation) if first-pass isolation was achieved in one or more ipsilateral PV pairs versus if achieved in neither pair (p = 0.0043).^38^ Greater success of first-pass PVI with less second-pass ablation may lead to less edema, shorter procedure times, and more durably ablated atrial myocardium. Further data are required to validate this endpoint as a surrogate for durability.

### Assessment for dormant pulmonary vein connection

Acute PVI during the index procedure is vital to prevent late PVR. The use of a waiting period and pharmaceutical adjuncts to reveal dormant PVR are important considerations.

PVR is seen more frequently the longer the wait after RFA; however, ablation with the strategy of a longer wait time to assess for PVR alone does not appear to improve AF recurrence rates.^39^ The use of adenosine to uncover dormant PVR has been well described in the literature. Specifically, adenosine acts to hyperpolarize the membrane potential through activating transient outward potassium currents (IK_Ado_) in PV cells to hasten spontaneous recovery of membrane potentials.

Recent work by Andrade et al. revealed 22.3% of patients with spontaneous PVR and 31.3% of patients with dormant (adenosine-unmasked) PVR existed in the RFA arm of their CIRCA-DOSE study; however, acute PVR (spontaneous or dormant) was not associated with a significantly increased incidence of recurrent tachyarrhythmia (hazard ratio: 1.47; 95% CI: 0.84–2.58; p = 0.16) or increased AF burden (p = 0.6088). However, when including patients from the cryoablation arm, acute PVR resulted in a higher AF burden and recurrent atrial tachyarrhythmia. Interestingly, in this study, spontaneous reconnection appeared prognostically more relevant than adenosine-induced dormant conduction.^40^ This study also suggested that optimizing the initial lesion set at first pass is key to ensure durability, with repeat ablation performed over prior suboptimal lesions being less effective than first-pass delivery in ensuring RFA-induced transmural contiguous necrosis.

### Baseline impedance and impedance drop

The interplay between resistance, power, and current is expressed by the following equation: power (*P*) = current squared (*I*^2^) × resistance (*R*). With power-controlled ablation, the current output constantly changes during RFA in response to measured impedance changes, with the alternating current output being only one factor influencing lesion size variability.^41^

Baseline impedance as a surrogate of “resistivity” of the energy delivery circuit has been demonstrated to better correlate with lesion size than impedance drop in ex vivo models.^42^ Baseline impedance at fixed power settings in this study was examined in 20 swine hearts using power-controlled irrigated catheters with a multistepped impedance load. Lesion dimensions were found to be highly variable, relating to differences in baseline impedance and current output. Lesions were deeper in locations where low impedance was noted relative to where higher resistance was present, consistent with a prior phantom model study.^43^

Histopathological data also demonstrate nonuniform tissue injury when RFA is applied in scarred myocardium versus when it is applied in normal myocardium, which is related to the heterogenicity of resistivity.^44^ This has implications for ablation outcomes—in particular, in patients with PeAF where atrial fibrosis is at a more advanced stage and the uniformity and transmurability of the lesion set can be compromised.

Impedance drop is a marker of tissue heating in response to RFA. As a predictor of the durability of a lesion using this parameter, there are recent data from 41 de novo PVI and follow-up procedures for PAF where reaching a local impedance drop of 17 Ω or greater in anterior/superior segments or 14 Ω or greater in posterior/inferior segments is predictive of chronic segment block.^45^ It is important to distinguish baseline impedance from an impedance drop, the latter of which reflects the effect of RFA on tissue rather than on the tissue properties itself.

The optimum baseline impedance with regard to safety and efficacy remains undefined but likely lies between 110 and 140 Ω based on the aforementioned animal and phantom studies, although values will vary according to the catheter used. Future studies with catheter technologies such as the IntellaNav StablePoint™ using the DIRECTSENSE technology in combination with the Rhythmia mapping system (Boston Scientific, Natick, MA, USA) examining local tissue characteristics and catheter stability in addition to local real-time impedance may help to create a more consistent lesion set, which may translate into achieving more durable results, to be assessed in randomized in vivo studies.^46^

## Catheter optimization

### Irrigated versus nonirrigated catheters

**Figure 2:**
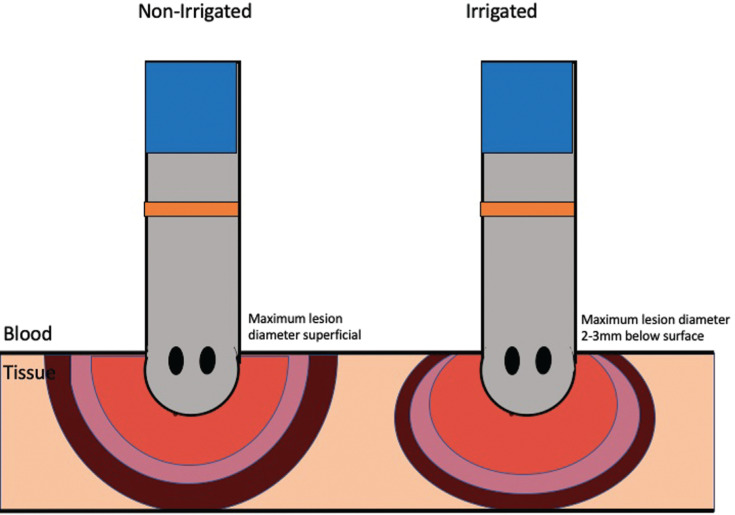
Comparison of irrigated versus nonirrigated catheter lesions.

The introduction of irrigated catheters has allowed for electrode cooling, limiting excessive heating of tissue and blood in close proximity to the ablation electrode, thus reducing thrombus formation and increasing lesion size.^47^ It is important to note that the surface lesion diameter decreases with use of irrigated catheters, with the maximum lesion diameter usually found 2 to 3 mm below the surface.^48^ This has implications for optimum ILD **(Figure 2)**.

Recent work presented at the European Society of Cardiology in 2020 by Morales et al. demonstrated that, in a population of 269 patients undergoing PVI for PAF and PeAF, the durability of the PVI lesion set was detected in 74% of participants treated with the ThermoCool^®^ SmartTouch™ Surround Flow catheter as compared with in 24% treated with the Thermocool^®^ Surround Flow catheter and 44% treated with the Thermocool^®^ SmartTouch™ catheter, respectively, with a mean time of 374 ± 331 days between redo ablation and the index procedure, highlighting the clinical utility of irrigation in combination with CF-guided RFA.^49^

Prior research has demonstrated increased RF current delivery when utilizing lower ionic irrigant, secondary to increased impedance surrounding the ablating electrode with decreasing dissipation of RFA into the irrigant. Recent preclinical work has demonstrated that the utility of low-flow, half-normal saline administered at 40 W for 30 seconds (10 seconds of which involved the ramp-up) and 8 to 14 g of CF led to similar lesions as those attained with normal saline in vivo, albeit the former were slightly deeper (p = 0.07) with an increased incidence of steam pop suggestive of increased tissue heating.^50^ This is in keeping with the findings of another recent preclinical study by Leshem et al.^51^ More adequately powered clinical studies analyzing lesion durability and safety are needed prior to determining the optimal choice of irrigant for RFA.

### Investigational radiofrequency catheters

One of the limitations in the use of current ablation catheter technology has been the narrow therapeutic window (low power leads to an incomplete effect, yet high power leads to tissue overheating) due to the high current density in standard ablation electrodes.^48^ The use of catheters with larger surface areas could help to deliver higher total energy amounts at a lower current density, widening the window of ablation.^52^ This certainly carries promise with the use of HPSD as described earlier.

A novel lattice tip catheter (Sphere-9; Affera, Inc., Watertown, MA, USA) uses multiple surface thermocouples and consists of a compressible spheroid-shaped, 9-mm in diameter lattice electrode tip delivering temperature-controlled, irrigated RFA. It creates wide, uniform lesions by virtue of its wide thermal footprint and ease of contiguity of the lesion set due to the lattice tip design. As a result, a smaller number of lesions are required relative to during traditional RF PVI to achieve isolation, with a shorter ablation time. A recent study has reported promising outcomes of durability in a small prospective, single-arm, first-in-human study. Among 65 patients undergoing PVI, protocol-mandated remapping at approximately three months occurred in 27 patients, with 26 of these 27 participants (99.1%) demonstrating durable PVI. All roof lines and 10 of 11 mitral isthmus lines also remained blocked. This translated clinically to a rate of freedom from atrial arrhythmia of 94.4% at a median follow-up of 270 days.^53^

Data from the ACT DiamondTemp Temperature-controlled and Contact Sensing Radiofrequency Ablation Clinical Trial for Atrial Fibrillation (TRAC-AF), a single-arm, prospective study that looked at the use of a diamond-tip, closed-loop irrigated catheter using integrated thermocouples, also demonstrated favorable durability at three months (84.8%) in a small number of patients (n = 23) undergoing repeat procedure following PVI (total n = 70 patients) for PAF.

### Unipolar electrogram morphology changes during pulmonary vein isolation

Interestingly, achieving real-time elimination of the negative component of the unipolar atrial electrogram during PVI may represent transmurability and constitutes an interesting endpoint that may be targeted to achieve durability. In vivo studies have demonstrated necrosis and transmurality (although potentially reversible) when RFA is terminated five seconds or more after the R-wave morphology changes, with greater collateral damage to the surrounding organs when RFA is terminated 10 seconds or more after this point in time.^54^ RFA-guided PVI by this approach, in conjunction with CF-irrigated catheters, was examined in a study of 250 consecutive PAF patients in the UNIFORCE study. Eleven percent of patients at two years had experienced AF recurrence, with 25 of 28 patients with recurrence demonstrating PVR.^55^ Outcomes have been encouraging when using this approach in other studies.^56,57^

### Pace-capture ablation

A method to search for gaps along the ablation lesion set line has been explored where pacing at a high output is used along the delivered lesion sets with RFA delivery at sites of capture.^58^ The adjunctive use of pace-capture assessment along the delivered lines of RFA lesions has been explored in adjunct to bidirectional PV block versus the latter assessment alone and, whilst a pace-capture technique results in a longer procedure, there appears to be significantly less AF detected during postprocedure monitoring with this approach.^59^

### Direct lesion-formation observation

The search for a means of real-time lesion visualization and assessment remains ongoing, with multiple modalities being explored at present.

The use of real-time MRI-guided RFA is yet to be validated in a clinical setting. Edema by virtue of T2-weighted imaging and necrosis by late gadolinium enhancement (LGE) after catheter ablation can provide information regarding the quality and transmurability of the lesion set. Additionally, this technology can assist with improved stability during ablation and real-time soft-tissue visualization during RFA.^60^ However, many limitations with this approach currently exist, including T2-weighted imaging being a poor predictor of chronic lesion volume,^61^ LGE use requiring up to one hour to fully enhance, and atrial arrhythmias causing difficulties in ECG gating. Device compatibility, the availability of MRI technology, and the use of MRI-compatible ablation equipment are also other considerations that much be addressed. Although MRI-guided ablation of AFL has been encouraging, the technology remains to be validated in the setting of more complex atrial arrhythmias. Further work is required in this field, including the gathering of long-term clinical data, before it can be embraced in routine practice.

Real-time imaging with nicotinamide adenine dinucleotide (NADH) fluorometric imaging in assessing catheter–tissue contact and predicting lesion depth as a correlate of NADH signal drop holds interest, having already shown promise in animal models,^62^ and may translate to more durable lesions, although clinical studies are warranted. Optical methods such as near-infrared spectroscopy and polarization-sensitive optical coherence tomography are also being explored for real-time lesion monitoring, albeit with limitations regarding tissue depth assessment.^63^ Dual-wavelength photoacoustics holds promise in real-time monitoring and depth-resolved lesion continuity assessment, with preclinical ablative research in porcine tissue demonstrating favorable results where photoacoustic-enabled RFA catheters can ablate and illuminate the myocardium with photoacoustics signals received by an intracardiac echography catheter.^64^ However, this technology again remains to be assessed in a clinical setting.

### Stability

The widespread adoption of electroanatomical mapping (EAM) has greatly aided the localization and stability of catheter manipulation within the atrial chambers. Other parameters used to improve catheter stability include the following.

#### General anesthesia

GA leads to minimization of patient movement and mapping system stability, which should lead to the creation of a more stable lesion set. A prospective randomized clinical trial from 2011 examined patients with PAF undergoing PVI with GA versus conscious sedation, with all patients undergoing a repeat procedure if they experience clinical recurrence of an atrial arrhythmia. In addition to demonstrating increased freedom from AF/AT in the GA arm, the study also showed reduced rates of PVR at repeat procedure in this group—that is, in 19% of patients who were administered GA versus 42% of those undergoing conscious sedation during the procedure.^65^ Follow-up assessments were performed in this study at three, six, nine, and 12 months after the procedure, involving a cardiology evaluation, 12-lead ECG, and seven-day Holter monitoring. In the recent PRECEPT study described earlier, 95.4% of patients were ablated under GA with a favorable rate for clinical freedom from atrial arrhythmias.

#### Ventilation strategies during general anesthesia

Instability due to respiratory variation during RFA application proves to be a troublesome barrier to contiguous effective lesion delivery, due to its impediment of catheter stability and CF.^66^

High-frequency jet ventilation (HFJV), which effectively eliminates thoracic movement, has been demonstrated in a study by Sivasambu et al.^67^ to significantly improve the mean force variability index relative to standard ventilation (p < 0.001), with improved maintenance of CF of less than 5 g during lesion delivery (79.8% vs. 76.7%; p < 0.001) and reduced symptomatic arrhythmia recurrence as compared with in matched control patients. However, when assessing the outcomes of durability, Holter monitoring was not routinely performed in all cases, and this study did not describe the incidence of PVR where a repeat study occurred.

Alternative ventilation strategies such as episodic apnea^66^ and the use of high-frequency, low-volume ventilation with episodic increases in tidal volume if CO_2_ accumulates are other strategies that may increase catheter–tissue interface stability, particularly at areas such as the left atrial appendage/left PV ridge.

#### Steerable sheaths

Steerable sheaths offer increased stability and can influence CF. One study reported improved one-year freedom from AF when steerable sheaths, EAM, and HFJV were used as compared with in patients in whom all three elements were not adopted.^68^ During first-time ablation in patients with PeAF, steerable sheaths were found to increase the ablation CF, with WACA correlating with a decrease in segments chronically reconnecting (manual nonsteerable sheaths: 26.5%; manual steerable sheaths: 4.6%; p < 0.0005) at a median redo time of eight months from the index procedure.^69^

#### Pacing local or adjacent chambers for stability

Modulating the heart rate by pacing from the coronary sinus catheter or the right ventricular catheter can be adopted to improve catheter stability and optimize the lesion set and durability. Approximately 30% of CF variability can be attributed to systolic–diastolic heart motion.^70^ Aizer et al.^71^ demonstrated a reduction in catheter–tissue contact variability with a right ventricular pacing approach, thereby increasing the probability of achieving prespecified catheter–tissue contact endpoints, with a significant improvement in impedance reduction during ablation. These findings were uniform across all areas of the LA as well as the cavotricuspid isthmus. However, comparing this approach versus not pacing the adjacent chamber in terms of lesion durability has not been studied.

## Patient factors

**Figure 3:**
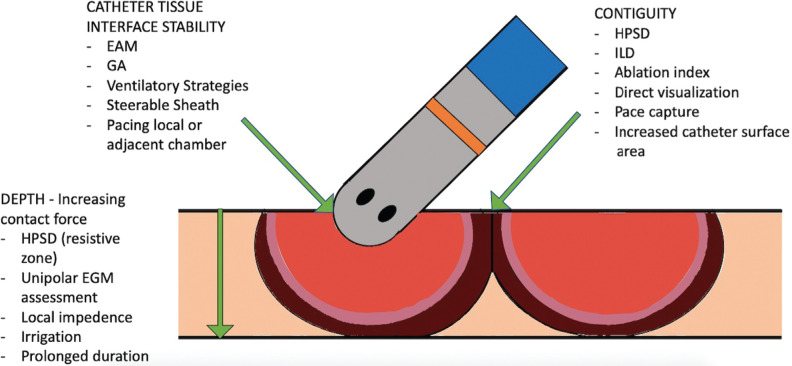
Parameters that impact the durability of radiofrequency lesions. EAM: electroanatomical mapping; EGM: electrogram; GA: general anesthesia; HPSD: high-power, short-duration ablation; ILD: interlesion distance.

Local tissue factors in the LA can also impact lesion depth and durability. We know from histological studies^72^ that the LA wall has a mean thickness of 1.5 to 2 mm, with a broader range from 0.5 to 4 mm found in men. As mentioned earlier, there is regional variation in thickness, with the thickest portion found in the left lateral ridge.^32^ This needs to be considered when selecting RFA parameters and counterbalancing adjusting settings to achieve transmurability versus the risk of steam pop and damage to surrounding structures. As previously mentioned, the heterogeneity of the atrial wall where a scar occurs also triggers imbalances in local impedance, leading to difficulty in conducting consistent transmural PVI,^44^ a finding that has implications for long-standing PeAF and diseased atria such as that found in patients with atrial amyloidosis **(Figure 3)**.

## Looking to the future

The pursuit of reproducible, durable atrial ablation is an essential struggle. Optimizing RFA delivery to achieve transmurability whilst avoiding collateral damage requires ongoing clinical randomized studies with appropriate endpoints of durability assessment (with invasive remapping at this current juncture).

Of the alternative future methods of ablation, pulsed-field ablation appears to hold great promise, having demonstrated impressive durability in a small study of PeAF patients (n = 25),^73^ with invasive remapping demonstrating 96% durability of PVI and 100% durability of posterior wall isolation, after showing impressive three-month durability of PVI in PAF (100%) also by invasive remapping.^74^ PFA, in particular, appears to offer an exciting capability to achieve transmurability with avoidance of collateral damage to the phrenic nerves and esophagus due to its myocardial selectivity.

## Conclusion

There exist a multitude of factors—both patient- and non–patient-related—that influence RFA durability **(Table 1)**. Optimizing these parameters is essential to ensure effective long-term results in our management of AF patients. Novel technologies such as the lattice-tip catheter and PFA, in particular, show exciting promise in achieving PVI durability with effective transmurability and reasonable safety in early studies. This review summarizes the multifaceted parameters that must be considered in delivering an optimized ablation set with the use of RFA.

## Figures and Tables

**Table 1:** Synthesis of Strategies That May Be Applied by Operators to Optimize Ablation Efficacy

Ablation Strategy	Mechanism	Supporting Studies
Depth and lesion quality		
Optimizing contact force	Optimizing contact between the catheter tip and the target tissue	TOCCASTAR,^23^ SMART-AF,^24^ EFFICAS 1 and 2,^27,28^ and PRECEPT^30^
High power, short duration (resistive zone)	Increase zone of resistive heating, wider lesion also improves contiguity, reduction of edema; energy (J) = power (W) × time (s)	Winkle et al.,^17^ Bunch et al.,^18^ and Yavin et al.^19^
Unipolar electrogram assessment	R-wave morphology change may represent lesion transmurability	UNIFORCE^55^
Baseline local impedance	Local impedance is a surrogate of tissue “resistivity”; adjusting the output in response to baseline impedance appears to improve lesion size and depth	Barkagan et al.^42^ and Bhaskaran et al.^43^
Impedance drop	Correlates with tissue heating; real-time assessment may improve delivery of an efficient lesion set	Bolao et al.^45^
Irrigation	Increases lesion depth, optimizing the lesion set	Morales et al.,^49^ Tschabrunn et al.,^50^ and Leshem et al.^51^
Pulsed-field ablation	Myocardial selectivity enables improved lesion quality	Reddy et al.^74^
Contiguity		
Interlesion distance	Eliminates gaps in the lesion set	CLOSE^32^
Ablation index, force–time integral	Objective lesion representation allowing optimization of power, contact force, stability, and time	CLOSE,^32^ CLOSE to CURE,^34^ PRAISE,^33^ and VISTAX^35^
Direct visualization (eg, MRI, NADH fluoroscopy, NIRS, PS-OCT, dual-wavelength photoacoustics)	Possibly improves catheter–tissue contact and depth	Currently being explored in a clinical setting
Pace capture	Identifying gaps in the lesion set	Steven et al.^59^
Increased surface area catheter (eg, lattice-tip catheter)	Wider thermal footprint with reduced current density improves contiguity	Reddy et al.^53^
Intraprocedural assessment for incomplete lesion set		
Adenosine administration	Hyperpolarization of membrane potential to hasten spontaneous recovery of membrane potentials and identify gaps in the lesion set	Andrade et al.^40^
First-pass isolation	Isolation of PV on first encirclement without the need for repeated lesions at gaps likely results in less edema and a more necrotic and durable lesion set	Osorio et al.^38^
Stability		
General anesthesia	Reduction in patient movement improving stability	Di Biase et al.^65^
High-frequency jet ventilation	Elimination of thoracic movement improves stability	Sivasambu et al.^67^
Steerable sheath	Reduction in variability of catheter movement	Ullah et al.^69^
Pacing local or adjacent chamber	Stabilizes cardiac motion improving stability	Aizer et al.^71^

MRI: magnetic resonance imaging; NADH: nicotinamide adenine dinucleotide; NIRS: near-infrared spectroscopy; PRAISE: Prospective Randomized Amlodipine Survival Evaluation; PRECEPT: Prospective Review of the Safety and Effectiveness of the Thermocool^®^ SmartTouch™ Surround Flow Catheter Evaluated for Treating Symptomatic Persistent Atrial Fibrillation; PS-OCT: polarization-sensitive optical coherence tomography; PV: pulmonary vein; TOCCASTAR: TactiCath™ Contact Force Ablation Catheter Study for Atrial Fibrillation; VISTAX: Evaluation of Ablation Index and VISITAG™ (ABI-173).
